# The Zoetrope effect in phage evolution

**DOI:** 10.3389/fmicb.2026.1856757

**Published:** 2026-07-20

**Authors:** Francisco Rodriguez-Valera, Ana-Belen Martin-Cuadrado

**Affiliations:** 1 Universidad Miguel Hernández, Campus de San Juan, Alicante, Spain; 2 Universidad de Alicante, Alicante, Spain

**Keywords:** arms-race, evolutionary stasis, long-term stability, phage evolution, trench warfare, Zoetrope effect

## Introduction

The Red Queen hypothesis has long dominated host-virus narrative frameworks: viruses and their bacterial hosts are locked in an unending evolutionary race, each adaptation met by a counter-adaptation, driving rapid genomic change (Stern and Sorek, 2011; Dennehy, 2012; Hampton et al., 2020). The extraordinary genetic diversity observed among natural phage populations—the so-called “paradox of viral diversity” (Weinbauer, 2004; Paez-Espino et al., 2016)—has reinforced this view, often being cited as evidence for continuous arms races. Although host adaptation driven by Red Queen dynamics is an undeniable process—well illustrated by the evolution of SARS-CoV-2 during the COVID-19 pandemic—our point is that this represents an atypical scenario rather than the baseline for well-adapted host-parasite interactions.

The “Zoetrope effect” revisited here (Rodriguez-Valera et al., 2014) offers a contrasting framework: the apparent diversity and seeming turnover revealed by genomic diversity snapshots may simply be successive frames of a stable system. Much like the nineteenth-century zoetrope animation, where static images produce the illusion of motion, the array of phage lineages detected in marine, freshwater, or host-associated ecosystems may represent multiple coexisting clones already in equilibrium with their hosts. In this view, evolutionary stasis (low in long-term directional change)—driven by ecosystem-level selection, coupled to finely tuned molecular co-adaptations—might be the rule for many abundant phage lineages.

Rather than endless genetic arms races, what we observe may be a meta-stable mosaic: high in concurrent clonal diversity, yet low in long-term directional change i.e., trench warfare (see below). This reframing invites us to also reconceive the unit of selection: not isolated phages chasing hosts, but phage-host consortia inhabiting high adaptive peaks. The persistence of virtually identical phage genomes across vast spatial and temporal scales supports this reinterpretation, challenging the assumption of constant rapid evolution and pointing instead to long-term ecological success through genomic conservation.

## Experimental evolution

A classical study in which phage lamba adaptation to its host (*Escherichia coli* B) by modifying the range of targets is provided in Meyer et al. (2012). In homogeneous pure cultures, bacteria often become resistant by losing or modifying the phage receptor, leaving little scope for continued coevolution. True reciprocal coevolution is thus rarely found in laboratory systems, where a common outcome is “host victory” without detectable phage counter-adaptation. This limitation was highlighted early by Lenski and Levin (1985), who showed experimentally that arms-race dynamics are strongly constrained in chemostats: resistant bacteria persisted indefinitely without sustained reciprocal adaptation. They argued that bacteria-phage coevolution is constrained by an asymmetry in evolutionary potential: bacteria can resist through multiple routes (receptor modification or loss), while phage counter-adaptation is limited to modified target binding, and the estimated waiting time for host-range mutants in phages, such as T4, exceeded 7 years.

In studies of experimental evolution (in which adaptation of resistance and infectivity has been detected over just a few generations; Buckling and Rainey, 2002; Brockhurst et al., 2007), hosts are more resistant to contemporary phages than ancestral strains indicating co-evolution (Gandon et al., 2008). These experiments seem to indicate that the host range of phages is very variable. However, there exists a trade-off between fitness and the breadth of parasite infectivity or host resistance. The fitness cost associated (for example nutritional requirements of the host) to these changes, is an important issue that is difficult to evaluate in pure cultures in which a single strain is challenged with a single phage.

With a more realistic set-up, Koskella and Parr (2015) studied coevolution phage-bacterium by direct isolation from inside leaves of the chestnut tree. Using a time-shift experiment with both sympatric and allopatric phages from either contemporary or earlier points in the season, they demonstrated that bacterial resistance is higher against phages from the past, regardless of spatial sympatry or how earlier in the season phages were collected. On the other hand, future bacterial hosts were more resistant to both sympatric and allopatric phages than contemporary bacterial hosts.

These studies therefore suggest not that phage host range is universally unstable, but that it can be highly labile under particular ecological circumstances (Scanlan et al., 2015). In some cases, shifts in infectivity can be linked to single mutations in phage receptor-binding proteins or in the corresponding host surface target (Drexler et al., 1989). Yet such apparently simple evolutionary changes are often associated with pleiotropic costs. For phages, a mutation that improves binding to one receptor variant may reduce performance on other hosts, thereby further narrowing the range of susceptible cells. For bacteria, modification or loss of the target receptor can have much broader physiological consequences, affecting nutrient uptake, cell-envelope properties, cell–cell interactions, and other components of ecological performance (Moller et al., 2019; Urvoy et al., 2026). Such trade-offs are difficult to evaluate in pure-culture systems involving only one bacterial strain and a single phage, where many of the ecological costs of resistance and infectivity remain hidden (Koskella and Brockhurst, 2014; Castledine et al., 2022). Laboratory experiments typically involve single bacterial strains challenged with single phages under homogenized conditions—scenarios that eliminate spatial refugia and constrain evolutionary options. There is a fundamental asymmetry: bacteria can survive without phages while phages cannot survive without their bacterial host.

## Evidence for long-term stability

Multiple studies report striking temporal and spatial stability in phage populations across marine, freshwater, terrestrial, polar, host-associated, industrial, and laboratory settings. In Table 1, we have recorded some cases of phage genomes recovered from distant times and places that reflect high genomic conservation (identity levels ranging from 97.7 to 100% nucleotide identity). One of the most remarkable examples is *Mushuvirus mushu* (Rozwalak et al., 2024), where a phage genome recovered from ~1,300-year-old human paleofeces shares 97.7% ANI with present-day gut phages from another continent. Short and Suttle (2005) recovered “indistinguishable” cyanophage g20 sequences (>99% identity) from the Gulf of Mexico, South Pacific Ocean, an Arctic freshwater cyanobacterial mat, and Lake Constance, despite major differences in salinity, temperature, and geography. The core genome of some cryoconite phages was recovered al 99% identity at locations at Svalbard and Greenland (Bellas et al., 2020), while some particularly variable features, such as the tail-fiber gene, were found identical at both locations (Rodriguez-Valera and Bellas, 2025).

**Table 1:** Representative examples of phage and archaeal virus genomic stability across environments, time, and geographic distance.

Phage/virus	Environment	Distance/time gap	Identity^a^	References
*Vibrio crassostreae* virulent phages	Pacific oyster farms, France	Same location; 4 years; >20 years (prophages)	100% ID	Liang et al., 2026
*Cutibacterium acnes* phage	Human skin (471 facial metagenomes)	>10 human subjects	Conserved defense mechanisms in host	Tripp et al., 2025
*Mushuvirus mushu*	Human paleofeces vs. present-day gut	1,300+ years; different continents	97.7% ID	Rozwalak et al., 2024
*Microcoleus* cyanophages (jumbo)	Freshwater benthic mats, Canada vs. California	~5,000 km cross-continental	Numerous gene-orthologs with high identity	Valadez-Cano et al., 2023
Gut Caudoviricetes	Human gut (338 individuals)	4 years	Stable composition	Gulyaeva et al., 2022
T4 phage laboratory stocks	Laboratory sublines, multiple facilities	48 years	>99% similarity	Subedi and Barr, 2021
Agricultural slurry virome	Agricultural slurry/manure	5 months	55% of vOTUs in all samples	Cook et al., 2021
Cryoconite hole phages	Arctic, Svalbard-Greenland cryoconite holes	Large distances; several years	~99% ID core Identical tail-fibers	Bellas et al., 2020 Rodriguez-Valera and Bellas, 2025
*Streptococcus thermophilus*	Same dairy processing facilities	Extended periods	Nearly unchanged genotypes	Szymczak et al., 2019
Marine phage MAVGs	Global ocean marine viromes (145 samples)	Years appart (2009-2013)	~99% ID	Gregory et al., 2019
crAssphage	Global human gut; non-human primates	Ancient host association	Phylogeographic clustering; colinear primate relatives	Edwards et al., 2019
Mediterranean marine phage MAVGs	Mediterranean Sea	Multi-year; varying depths	>99% ID	Lopez-Perez et al., 2017
Human gut viral contigs	Human gut (adult longitudinal)	2.5 years	80% of 478 contigs persisted	Minot et al., 2013
Mediterranean marine phages	Mediterranean deep chlorophyll maximum	Multi-year	100% ID	Mizuno et al., 2014
*Staphylococcus pasteuri* vs. *Bacillus cereus/B. thuringiensis* phages	Antarctic soils vs. China	Geographically distant	99% ID	Swanson et al., 2012
*Synechococcus* cyanophages	Coastal New England	Same location; 15 years	>98% ANI	Marston and Martiny, 2016
*Mollivirus sibericum* vs. *M. kamchatka*	Ancient permafrost vs. modern subarctic soil	~30,000 years	92% average protein identity	Christo-Foroux et al., 2020
*Fuselloviridae* (SSVs)	Hot springs: Kamchatka, Yellowstone, Japan, Iceland, Philippines	Continental-scale separation	highly colinear core genome	Wiedenheft et al., 2004; Redder et al., 2009; Zhang et al., 2020
Marine MAVs	Marine (Surface), California	53 monthly samples over 5 years	97% of contigs were detected in each sample; >99% ID to previously described phages	Ignacio-Espinoza et al., 2020
*Pithovirus sibericum* vs. *P. massiliensis*	Siberian permafrost vs. modern sewage	~30,000 years	93.5% ortholog content; 83.6% ANI	Levasseur et al., 2016
Prophages in *Escherichia and Salmonella*	Bacterial chromosomes	Millions of years	Conserved integration hotspots; purifying selection in core genes	Bobay et al., 2013, 2014
Viral populations in Lake Tyrrell	Hypersaline, archaeal-dominated lake, Australia	4 days to ~3 years	91–100% ID	Emerson et al., 2012
Cyanophage g20	Gulf of Mexico, South Pacific, Arctic freshwater mat, Lake Constance	Cross-biome	>99% ID	Short and Suttle, 2005

ID, nucleotide identity.

^a^“Identity” column reflects a selected subsets of data (phages) described in the referenced original papers.

Marine systems provide particularly strong support for long-term persistence. Several studies have described phage populations that remain stable across many years (Marston and Martiny, 2016), and even reports exist of nearly identical phage genomes recovered from distant oceanic locations (Rodriguez-Valera et al., 2014). In the Mediterranean, several metagenome-assembled viral genomes exceeded 99% identity across different years and depths, with core genomic regions recruiting reads at close to 100% identity (Mizuno et al., 2014; Lopez-Perez et al., 2017).

## Defense systems as modulators of phage activity

A major argument for ongoing arms races is the remarkable diversity of phage-resistance systems encoded in prokaryotic genomes, and the presence of resistance-compensating genes in some phages. The genomic investment in such systems is indeed suggestive of intense host-phage conflict. However, these mechanisms need not be interpreted exclusively as weapons in perpetual warfare; they may also function as modulators or feedback regulators of phage activity.

Consider CRISPR-Cas: a phage recognized by a spacer cannot successfully infect the host, but if phage DNA has entered the cell, genetic recombination may still occur, allowing phage gene variants to persist (Hille et al., 2018). Similarly, abortive infection and toxin-antitoxin systems can prevent phage propagation while releasing cellular contents—including phage DNA—into the environment, potentially facilitating gene persistence and recycling. In a way, CRISPR might just provide an alternative way for host-phage interaction, like lysogeny. In addition, these mechanisms may serve primarily to modulate phage activity within stable bounds, rather than to drive continuous escalation.

The converse is equally important: many bacterial defense systems are themselves encoded by prophages and appear to function by limiting superinfection or controlling coinfection dynamics. This suggests that part of what is often interpreted as host defense may instead reflect phage-mediated regulation of phage–phage interactions within the host. Consistent with this, temperate phages often show long-term genomic associations with their hosts. Prophage integration hotspots are highly conserved across *Escherichia* and *Salmonella*, indicating that prophage–host associations can remain stable over millions of years (Bobay et al., 2013). Moreover, vertically inherited prophage elements that may predate the *Escherichia-Salmonella* divergence show signatures of purifying selection even in core phage genes (Bobay et al., 2014). Together, these observations support the view that many defense systems are not merely antagonistic devices, but components of a stable regulatory framework governing phage activity, lysogeny, superinfection, and gene flow.

## Discussion

The collected evidence supports the hypothesis that Red Queen dynamics may represent an evolutionary capacity for change rather than a permanent state. Laboratory experiments, with their high-density, well-mixed, resource-rich conditions, appear to exaggerate the speed and intensity of phage–host coevolution relative to what occurs in natural environments. Natural systems—human skin (*Cutibacterium acnes*–phage), the gut virome (crAssphage), acidic hot springs (*Fuselloviridae–Sulfolobus*), hypersaline environments (Lake Tyrrell), and the oceans (cyanophages)—provide clear examples of genomic conservation and evolutionary stasis. Successful phage lineages occupy high adaptive peaks where genetic change is more likely to reduce than enhance fitness. In fact, some bacterial pathogens and their (plant) hosts have also been proven to be in a kind of equilibrium referred to as “trench-warfare” (Stahl et al., 1999). In this case, advances and retreats of resistance-allele frequency maintain variation for disease resistance as a dynamic polymorphism in which host and parasite can persist indefinitely.

This reframing has several implications. First, it invites reconceptualization of the unit of selection: not isolated phages chasing hosts, but phage-host consortia inhabiting stable ecological niches (Rodriguez-Valera et al., 2009). Second, it implies that extensive host phage-resistance mechanisms may function primarily as modulators rather than weapons in perpetual conflict. Third, it indicates that laboratory evolution experiments, while valuable, may not accurately represent natural dynamics in spatially structured, diverse communities.

Critical questions remain. What determines whether a phage lineage enters arms-races vs. trench-warfare? How does spatial structure in natural environments mediate these dynamics? Can we predict which phage-host systems will exhibit stability vs. turnover? Long-term metagenomic time series, global phage tracking studies, and analysis of ancient viral DNA will be essential for addressing these questions. The paradox of bacterial resistance mechanisms also deserves particular attention: the genomic investment in CRISPR-Cas and other defense systems appears inconsistent with stable phage/host populations, yet these mechanisms may serve regulatory rather than defensive roles. Even failed infections may facilitate horizontal gene transfer, maintaining genetic connectivity between phage and host populations.

Ultimately, the Zoetrope effect invites a philosophical shift (Heraclitean vision vs. Parmenides). The widespread acceptance of the arms-race model might be just an example of anthropocentric bias. Evolutionary stasis can be as ecologically dynamic as rapid change. The persistence of ancient, highly adapted phage lineages across millennia and oceans testifies not to evolutionary stagnation but to the profound success of optimization. In the largest genetic reservoir on Earth, stability may be the rule rather than the exception.
